# Review of "Cardiac Fibrillation-Defibrillation: Clinical and Engineering Aspects", by Max E. Valentinuzzi

**DOI:** 10.1186/1475-925X-10-67

**Published:** 2011-08-02

**Authors:** Walter H Olson

**Affiliations:** 1 Medtronic, Inc. MVN41, 8200 Coral Sea Street NE, Mounds View, MN 55112, USA

## 

This book [1] focuses on fibrillation and defibrillation with extensive historical material, qualitative descriptions of fibrillation mechanisms, defibrillation phenomena, defibrillator devices, detection algorithms, electrodes, pastes, safety, efficacy and theoretical models. The author, Max E. Valentinuzzi from the University of Buenos Aires, Argentina is a biophysist who studied cardiovascular physiology with Hebel Hoff and Leslie A. Geddes at Baylor in Houston in the early 1970s [2].

The "seed" for this book was the author's 25 year old small book on Cardiac Fibrillation-Defibrillation written in Spanish that was "fully updated and revamped". The style, descriptions and old Physiograph ECG recordings remind me of early Geddes texts that were very qualitative. Extensive recent research from the laboratories of Ray Ideker, Jose Jalife and Igor Efimov is underrepresented. The reader will find many interesting and colorful editorial commentaries and speculations.

Chapter 1 defines fibrillation as "asynchronic", "uncoordinated" and "chaotic". Epicardial or body surface maps of VF would have helped the reader understand these concepts. The mechanisms of fibrillation are described as "reentry", "multiple ectopic foci", and "rotor or vortex". Atrial fibrillation and flutter are described within several chapters, but recent ablation therapies for AF and VT/VF are only briefly described.

Chapter 2 on defibrillation covers a very broad range of topics including history, chemical defibrillation, transthoracic and transventricular electrical defibrillation, the probabilistic nature of defibrillation, physiologic and technologic variables with emphasis on critical mass, ionic concepts, defibrillation thresholds, defibrillation dose, and a brief section on cardiopulmonary resuscitation.

Chapter 3 on defibrillators focuses on historical aspects, simplistic block diagrams, capacitor discharge waveforms and biphasic truncated exponential waveforms. A section on implantable cardioverter defibrillators (ICD) contains several erroneous and misleading statements such as "the device detects an irregular signal". In fact, all transvenous ICDs use bipolar local intracardiac electrograms to detect high rates during VF regardless of "irregularity" in electrogram amplitudes or cycle lengths. I asked a colleague who is more knowledgeable about transthoracic fibrillation and defibrillation to look at the book and he said "those sections weren't particularly accurate or informative." The books author repeatedly transitions between external defibrillators and implantable cardioverter-defibrillators without warning and no apparent appreciation for the vast differences. Wearable defibrillators and subcutaneous ICDs are ignored.

Chapter 4 on ventricular fibrillation detection was written by coauthor Eric Laciar Leber beginning with performance definitions for sensitivity, specificity, positive predictivity and receiver operating curves. The seven VF detection algorithms described have not been used in real devices because they exceed real-time computational limits or have medically unacceptable performance. ICD manufacturers have found practical implementations for wavelets and amplitude templates for signal morphology.

Chapter 5 on electrodes and pastes describes the electrode-electrolyte interface impedance using electric circuit models. The interface is correctly described as primarily resistive for defibrillation shocks. The critical topic of defibrillation electrode positions on the thorax for transthoracic defibrillation is described only briefly without diagrams. This chapter has a discussion of basic medical electrical safety, but no description of the micro-shock concept where fibrillation is induced at much lower current levels when there is a conductive pathway to the myocardium, such as a temporary pacing wire. Information on electromagnetic interference and standards is brief and outdated. The most practical ICD defibrillation vector that has been in use for many years, namely from the right ventricular coil electrode to the subcutaneous implanted can, is not mentioned anywhere in this book.

Chapter 7 on theoretical models written by coauthor Diego Gonzalez compares top-down versus bottom-up modeling strategies, then the Hodgkin-Huxley membrane model and various dynamic non-linear models that are minimally connected to cardiac or fibrillation concepts. The works of Poincare, van der Pol, Bonhoeffer, Winfree, FitzHugh-Nagumo, Keldermann and Weiss are described. Later in this chapter Simone Giannerini describes statistical models for complex systems.

The conclusions/discussion in Chapter 8 describes many topics: inflammation, torsades de pointes, atrial fibrillation, resuscitation, defibrillators, ICDs, non-linear dynamics, and the allometric law to relate the probability of fibrillation to body mass and the number of diseased ventricular myocytes using the ST segment of the ECG.

In summary, the strengths of this book are the historical material and its focus on fibrillation and defibrillation. The qualititative approach, internet citations and commentaries will interest some general readers. There is little that clinicians can apply to medical practice. Engineers will not be satisfied with the technical aspects of defibrillators and their design. Scientists and researchers in this field may find the text by Efimov, et al. [3] more useful.

## Competing interests

The author declares that they have no competing interests.

## Authors' contributions

WO is the sole author.

## Author information

Vice President, Emeritus, Ph.D., FHRS. LLC
